# Cariprazine as a treatment for negative psychotic symptoms in first-episode psychosis: case series

**DOI:** 10.1192/bjo.2022.56

**Published:** 2022-04-28

**Authors:** Arsime Demjaha, Eduardo Iacoponi, Lars Hansen, Pradeep Peddu, Philip McGuire

**Affiliations:** Department of Psychosis Studies, Institute of Psychiatry, Psychology & Neuroscience, King's College London, UK; and NIHR Maudsley Biomedical Research Centre, South London and Maudsley NHS Foundation Trust and King's College London, UK; Lambeth Early Intervention Service, South London and Maudsley NHS Foundation Trust, UK; Department of Psychiatry, Southampton University, UK; Psychosis Service, Coventry and Warwickshire Partnership NHS Trust, UK; Department of Psychosis Studies, Institute of Psychiatry, Psychology & Neuroscience, King's College London, UK; and NIHR Maudsley Biomedical Research Centre, South London and Maudsley NHS Foundation Trust and King's College London, UK

**Keywords:** Negative symptoms, cariprazine, first-episode psychosis, antipsychotics, novel central nervous system drugs

## Abstract

Negative psychotic symptoms are among the most disabling features of schizophrenia, and are strongly associated with relatively poor clinical and functional outcomes. However, there are no effective treatments for negative symptoms, and this represents a major unmet clinical need. Recent research has shown that negative symptoms are already present in many patients at illness onset. There is evidence that cariprazine may improve negative symptoms in patients with chronic schizophrenia. However, its utility in treating negative symptoms in the early stage of the disorder is unclear. Here, we report six cases of patients with first-episode psychosis who were treated with cariprazine.

Negative symptoms are among the most incapacitating features of schizophrenia.^1^ They contribute to impaired social functioning, which is particularly problematic in the early stages of the disorder,^2^ where it has a prevalence of 23–40%.^3^ At present, there are no effective treatments for negative symptoms,^4^ and this represents one of the most important unmet therapeutic needs in psychiatry.^1^ Recent clinical trials involving patients with chronic schizophrenia suggest that the novel antipsychotic cariprazine may be beneficial in the treatment of negative symptoms;^2,5^ however, the effectiveness of cariprazine on negative symptoms in patients with first-episode psychosis (FEP) has yet to be evaluated. Here, we describe a series of six patients with FEP that were treated with cariprazine in UK early intervention services (EIS). To the best of our knowledge, the cases described in this series provide the first indication that cariprazine may be effective in the treatment of negative symptoms in FEP.

We examined the clinical information of patients with FEP who were presenting with negative symptoms, including treatment response, based on rigorous clinical assessments and close observations by highly experienced EIS consultants. On qualitative exploration, our sample comprised five men and one woman, with a mean age of 29.5 ± 5.5 years (range 24–37 years). The mean dosage of cariprazine administered was 2.5 ± 0.77 mg/d (range 1.5–3 mg/d), with a time to response of 4.5 ± 2.3 weeks (range 1–8 weeks). Clinical and demographic characteristics of the sample are presented in Table 1. There was a clinically meaningful improvement in negative symptoms in four cases in which cariprazine was used as a monotherapy, and in one case when it was given as an adjunct to lurasidone. In one case, cariprazine had to be discontinued shortly after the start of treatment, because of a dystonic reaction.

**Table 1:** Clinical and demographic characteristics of six patients with first-episode psychosis who received cariprazine treatment

Patient	Age, years	Gender	Cariprazine dosage, mg/d	Adjunct antipsychotics	Time to response, weeks	Cariprazine side-effects	Negative symptoms at presentation	Clinical outcome
1	37	Male	3.0	None	5.0	Mild initial sedation	Social and emotional withdrawal, blunted affect, poverty of speech, loss of interests	Improved affect, emotional expression and spontaneity. Speech more coherent and spontaneous. Started attending programmed psychosocial activities
2	28	Male	1.5	Lurasidone at 55.5 mg/d	1.5	None reported	Amotivation, avolition, active and passive social avoidance, anhedonia	Stated that they were feeling much happier, had managed to cycle short distances, reinstated their internet connection and resumed gym attendance
3	29	Male	3.0	None	8.0	Akathisia (tolerable) at the higher dosage	Amotivation, avolition, emotional and social withdrawal associated with significant decline in functioning (unable to hold down a string of jobs and had given up playing the guitar)	Activities of daily living markedly improved. He started to engage with the psychological therapy and started attending group activities within the early intervention service team
4	35	Female	3.0	None	4.0	None reported	Blunted affect, anhedonia, amotivation associated with significant decline in functioning (required a lot of support from her partner to look after herself and their children)	Improvements in motivation and energy levels and was able to enjoy activities and care for her children. Her day-to-day functioning markedly improved
5	24	Male	3.0	None	4.0	Initial nausea	Amotivation, anhedonia, social withdrawal, alogia, poor self-care and blunted affect	Increase in energy levels, reignited interest in activities, particularly recording music online. His interactions with relatives also improved, as did his self-care and hygiene
6	24	Male	1.5	None	0.5	Acute dystonic reaction requiring treatment with intravenous procyclidine	Amotivation, anhedonia, reduced emotional expression	Did not complete treatment because of significant side-effects

## Ethics

Informed verbal consent was obtained by treating consultants and recorded in respective medical records.

Ethical approval is not required for case series.

## Discussion

Cariprazine is a dopamine D3/D2 potent partial agonist with a greater affinity for D3 than for D2 receptors and additional partial agonist activity at serotonin 5-HT1A receptors.^2,6,7^ Preclinical data suggest that antagonism at D3 receptors, preferentially expressed in the mesolimbic dopamine circuit,^8^ increases dopaminergic transmission in the prefrontal cortex,^9^ which could lead to an improvement in negative symptoms. Animal studies further indicate that cariprazine has anti-anhedonic and pro-cognitive effects.^10^ In clinical trials, it has been reported to have significantly greater efficacy for negative symptoms than risperidone in patients with chronic but stable schizophrenia,^2^ and aripiprazole in patients with an acute exacerbation of illness.^5^

In chronic schizophrenia trials of cariprazine, the maximum effect on negative symptoms was evident after 26 weeks of treatment.^2^ Here, a relatively quick time to cariprazine treatment response was observed, as is the case with other antipsychotics in FEP.^11^ In addition, our case series suggest that in early psychosis, lower dosages of cariprazine are required to achieve therapeutic effect than those reported in a clinical trial of patients with chronic illness (mean 4.2 mg/d),^2^ similar to other antipsychotics at early stages of illness.^12^ Interestingly, its concomitant use with lurasidone (see Table 1, patient 2) resulted in a much quicker response and at its lower dosage, a result which requires further evaluation in rigorous trials where cariprazine may be administered as adjunct treatment.

Although cariprazine was well-tolerated in five patients, one patient developed acute dystonic reaction (ADR). Partial dopamine agonists are associated with a low risk of extrapyramidal side-effects, as they do not completely antagonise dopaminergic activity in the nigrostriatal or tuberoinfundibular pathways.^13^ However, ADR following treatment with aripiprazole, another partial agonist, has been documented.^14^ Patients with FEP are more sensitive to the adverse effects of antipsychotics,^15^ and it is possible that the unexpected ADR in our patient was related to the early stages of the disorder and an observed general sensitivity to antipsychotic medication, including partial agonists.

To our knowledge, this is the first report of a case series in which cariprazine was used for the treatment of negative symptoms in FEP. Although the observations suggest that cariprazine may be useful for this indication and may alert clinicians to a novel, more effective treatment for negative symptoms, the findings are retrospective and involve a small number of patients. Large-scale, double-blind, randomised controlled trials in patients with FEP are required to formally investigate the efficacy of cariprazine in treating negative symptoms at the earliest stages of psychotic illness.

## Data Availability

Data that support the findings of this study are available from the corresponding author, A.D., upon reasonable request. The data are not publicly available due to containing information that could compromise the privacy of participants.
